# Supplementary material for: Super-resolution upgrade for deep tissue imaging featuring simple implementation
Source: Nat Commun. 2025 Jun 25;16:5386. doi: 10.1038/s41467-025-60744-y (PMC12198360; doi:10.1038/s41467-025-60744-y)
Supplement: Supplementary file 1 — Supplementary information [file 41467_2025_60744_MOESM1_ESM.pdf]

Super-resolution upgrade for deep tissue imaging featuring simple implementation: supplemental document

Patrick Byers[1,2], Thomas Kellerer[1], Miaomiao Li[3,4], Zhifen Chen[3,4], Thomas Huser[2], Thomas Hellerer[1*]

[1*]Multiphoton Imaging Lab, Munich University of Applied Sciences, Lothstraße 34, Munich, 80335, Germany.
[2]Biomolecular Photonics, Department of Physics, Bielefeld University, Universitätsstraße 25, Bielefeld, 33615, Germany.
[3]Department of Cardiology, German Heart Center, TUM University Hospital, TUM School of Medicine and Health, Technical University Munich, Munich, 80636, Germany.
[4]Deutsches Zentrum für Herz- und Kreislaufforschung (DZHK), partner site Munich Heart Alliance (MHA), Munich, Germany.

*Corresponding author(s). E-mail(s): hellerer@hm.edu;
Contributing authors: pbyers@hm.edu; thomas.kellerer@hm.edu; miaomiao.li@tum.de; chenz@dhm.mhn.de; thomas.huser@physik.uni-bielefeld.de;

1 Hardware synchronization

Fig. S1a depicts the hardware diagrams of the proposed setup. The microscope is controlled via a custom-written MATLAB script named LiL-SIM GUI. Trigger signals and voltage ramps are generated and directed to the hardware components of the microscope via a data acquisition (DAQ) card, which is linked to a breakout box with physical BNC connectors. The camera settings including frame exposure time, line exposure time of the rolling shutter and Lightsheet shutter mode settings are set by the open-source software Micro-Manager [1]. The timing diagram of the LiL-SIM setup is shown in Fig. S1b. The camera is triggered via an external TTL pulse, which starts the frame acquisition. Voltage ramps are generated with discrete increments, which leads to stepwise scanning via the non-resonant galvo-scanner. A flyback array is added at the end of each scan array to allow for smooth transition to the starting position. The individual voltage ramps are to enable phase shifting of the illumination pattern. After all corresponding images are recorded for a certain direction, ext. trigger 2 is applied to hardware trigger the rotation unit. This procedure is repeated until all images (usually 9 or 15 images) are acquired. The modification for hardware triggering the rotation mount specified in the components table can be found in the GitHub repository. Detailed wiring diagrams, technical notes and relevant code, as well as a parts list to rebuild the LiL-SIM setup are provided in the Github repository availabe under https://doi.org/10.5281/zenodo.15031580.

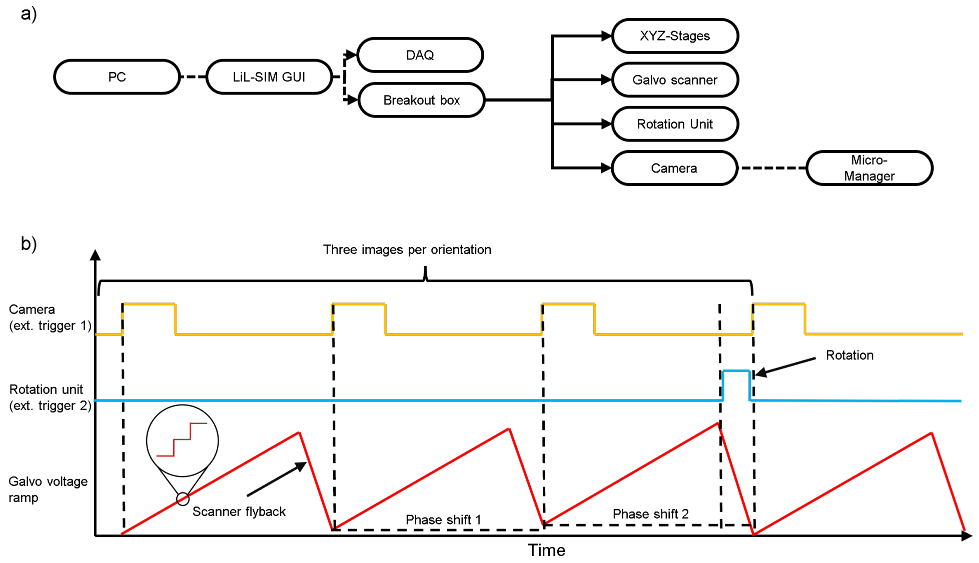

Figure S1 a) Hardware diagram of the LiL-SIM setup. The custom-written LiL-SIM GUI controls a DAQ card which is used for controlling translation stages, the galvo-scanner and the rotation unit. A frame trigger is provided to the camera via the LiL-SIM GUI while the camera settings are controlled in Micro-Manager. b) Timing diagram of the LiL-SIM setup for acquiring a LiL-SIM stack with three phases at a single orientation angle. The curves show the frame trigger provided to the camera (yellow), the trigger for the rotation unit (blue) and the galvo voltage ramp (red). Phase shifts of the individual illumination patterns are generated by a voltage offset supplied to the galvo-scanner.

2 Imaging routine of LiL-SIM

LiL-SIM requires digital post-processing to obtain a super-resolution image from the acquired images. The post-processing routine is shown in Fig. S2.

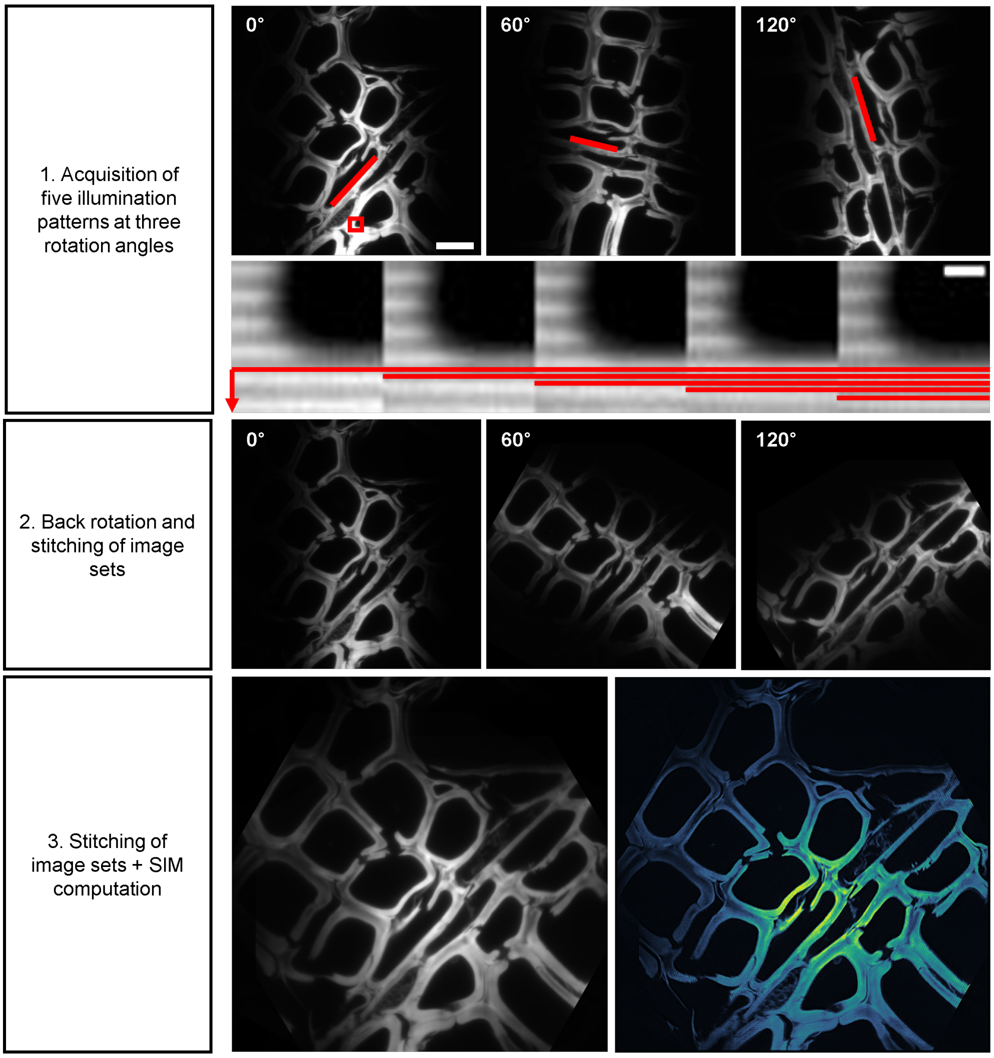

Figure S2 Imaging routine of LiL-SIM. Step 1: Five illumination patterns are acquired per rotation angle. The red bar indicates the rotation of the imaged structure. Insets show the shift of the scanned pattern by phase steps of 2pi/5. Step 2: Images are digitally back rotated corresponding to the rotation angle of the rotation mount. Step 3: Image sets are backrotated by a custom-written algorithm (explained in 15) to match the file format of fairSIM input data. For all images, the brightness has been adjusted to a saturation of 0.35. After reconstruction, we applied flat-field correction based on the recorded excitation matrices in thick fluorescent slides (see Fig. S13). Scale bars 10 µm, inset 500 nm.

3 Imaging parameters

The power in the BFP amounted to 200 mW which results in a peak intensity of 106 GW/cm^2 for all experiments. λ_{em} [nm] specifies the fluorophore emission wavelength. d_p [nm] specifies the pattern spacing. EBW = exposure band width in pixels. z_{Step} = step interval of the z-stacks. z_{range} = depth of the acquired volume (over a ROI of 1024x1024 pixels). t_{exp} = exposure time per line. t_{acq} = acquisition time for a single frame.

**Sample**	**Figure**	λ_{em} [nm]	d_p [nm], EBW	$z_{\mathrm{Step}}/z_{\mathrm{range}}$ [μm]	$t_{\mathrm{exp}}/t_{\mathrm{Aqc}}$ [ms]
**Manuscript**					
Fluorescent beads	1	525	350 / 7	-	5 / 955
Pinus radiata	2b, 2c	650	200 / 7	0.5/0-40	5 / 1670
Zebrafish	2d, 2f	550	350 / 7	0.5/0-80	5 / 955
Pinus radiata	3	650	300 / 7	0.5/0-40	5 / 1115
Heart muscle	4	650	350-400 / 7	0.5/0-70	5 / 955-835
**Supplementary**					
Pinus radiata	S2, S5, S6, S9, S10	650	350 / 7	0.5/0-40	5 / 955
Pinus radiata	S3, Video1	650	350 / 7	0.5/0-40	5 / 955
Pinus radiata	S10, Video1	650	200 / 7	0.5/0-40	5 / 1670
Pinus radiata	Video2	650	350 / 7	0.5/0-40	1 / 76
Argolight SIM slide	S4, S8	525	350 / 7	Var.	5 / 955
Fluorescent slide	S7, S12	525	Var. / 7	0.5 / 0-120	Var.

Table T1 LiL-SIM setup parameters.

**Sample**	**Figure**	λ_{ex} [nm]	**Power** [mW]	$z_{\mathrm{Step}}/z_{\mathrm{range}}$ [μm]	t_{Aqc} [ms]
**Manuscript**					
Pinus radiata	2a	800	7	0.2/0-40	2100

Table T2 PMT-2PM setup parameters.

4 SBR / SNR analysis

We compared the resulting SBR when either using point-detectors or spatial detectors (i.e. cameras). The SBR mainly depends on two properties: (1) the lateral and axial extent of the PSF and (2) the scattering properties of the specimen. In the case

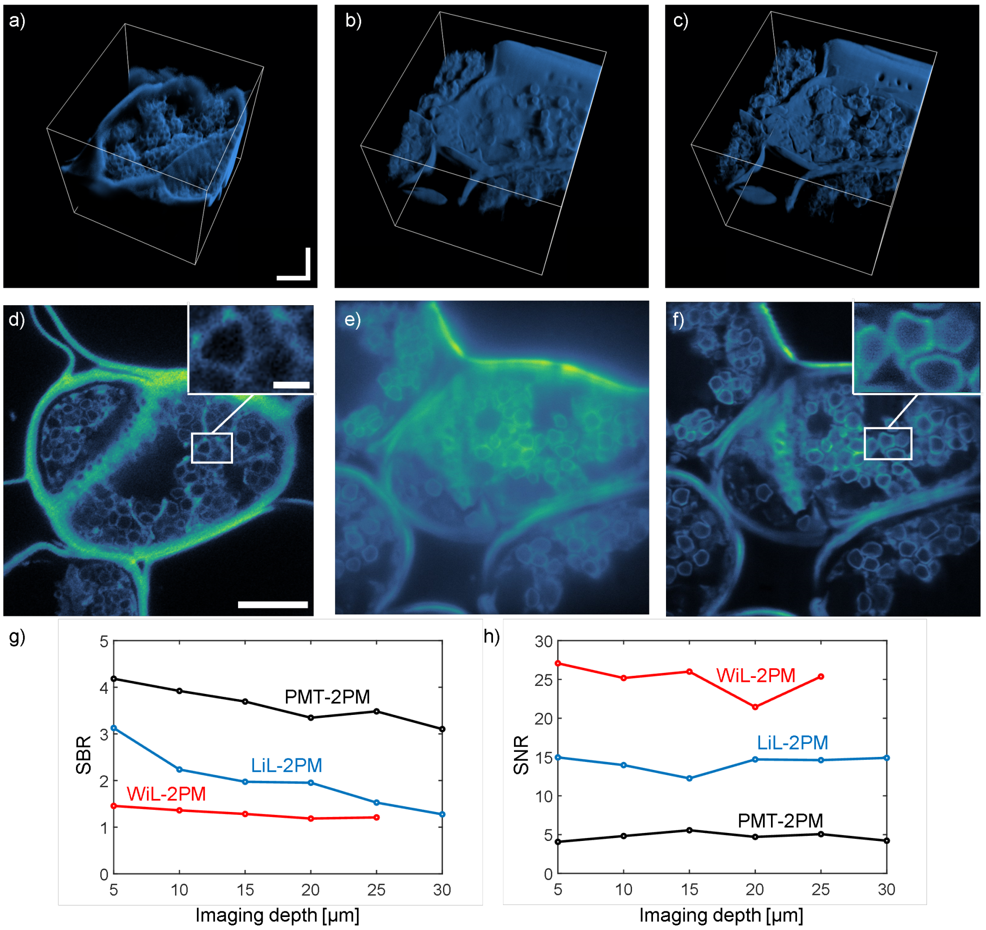

Figure S3 Impact on signal-to-background ratio (SBR) and signal-to-noise ratio (SNR) for different imaging modalities. a–c) 3D volume renderings of the same sample acquired with a) point-scanning setup with a photomultipler tube detector (PMT-2PM), b) line-scanning with a camera detector using the rolling shutter mode (WiL-2PM), and c) line-scanning with lightsheet shutter mode (LiL-2PM). Please note that a) was acquired with an entirely different setup than b) and c). d–f) Single-plane images showing vessel structures at an imaging depth of approximately 20 µm below the surface. g) Signal-to-background ratio (SBR) plotted as a function of imaging depth for the three configurations. The results demonstrate that PMT-2PM achieves higher SBR at greater depths than LiL-2PM and WiL-2PM. h) Signal-to-noise ratio (SNR) as a function of imaging depth. The signal-to-noise ratio is increased for camera-based detection due to the longer integration times per pixel. Experiments have been repeated (N = 3) times. Scale bars a-c) lateral and axial 10 µm, d-f) 10 µm, inset 3 µm.

of conventional spot-scanning 2P microscopy (including a point detector), the optical transfer function (OTF) of the system can be described as the autoconvolution of the 2P excitation OTF. Contrary, the system OTF of camera-based 2P microscopy is expressed as the convolution of the 2P excitation OTF and the 1P emission OTF. Consequently, the (axial) missing cone is more efficiently filled in the case of conventional point-scanning 2P microscopy, while camera-based 2P microscopy yields an increased lateral and axial extent of the system OTF, resulting in higher lateral and axial resolution. It is not straightforward to discuss a crossover point here, because the achieved optical sectioning fundamentally depends on the density and scattering properties of fluorescent structures contained in the specimen. Despite the fact, that the SBR of a point-detector based setup should always be increased compared to a camera-based setup, camera detection is mandatory in order to reconstruct SIM images. We used a commercially available multi-photon setup (Miltenyi Biotec, TrimScope Matrix) and set similar frame times and excitation wavelengths for the acquisition to allow for a comparison to the camera-based LiL-SIM system. However, the laser power is substantially lower when using point-excitation (i.e. 7 mW compared to 200 mW when using line-scanning with 1024 pixels per line). Fig. S3a compares the imaging modalities of a) point-scanning two-photon microscopy (PMT-2PM), where a photomultiplier tube is used as detector. Volume stacks of the same size, acquired in similar regions with LiL-2PM and WiL-2PM are shown in Fig. S3b-c, respectively. In Fig. S3d-f single planes with resin ducts, including xylem vessels are depicted in planes approximately 20 µm below the surface. We quantify SBR and SNR by evaluating the intensity inside xylem vessels (background) vs. the intensity of the vessel membrane (signal). This is achieved by measuring the signal intensity along the ring. Further, the background signal is evaluated by taking the mean of a 20x20 pixel area inside vessels. Ten vessels were evaluated per imaging depth and the SBR was evaluated starting at an imaging depth of 5 µm. SBR and SNR curves comparing the modalities are shown in Fig. S3g-h. The SBR of PMT-2PM is increased when using the commercial setup and decays for higher imaging depths. However, since the individual pixel dwell times are set substantially longer in a camera detector (1.16 µs vs. >= 5ms / pixel), the SNR is severely increased when using the camera modalities.

5 LiL-SIM with various objective lenses

LiL-SIM can be used with various objective lenses. We compared the achieved resolution with 100x / 1.49 NA, 60x / 1.27 NA, and 40x / 1.15 NA lenses by measuring line pairs with decreasing distance ranging from 390 nm to 0 nm (shown in Fig. S4). Fig. S4a compares the line pairs of LiL-SIM and the deconvolved averaged image set when using the 100x objective lens. It can be clearly seen that LiL-SIM improves the resolution significantly, which allows for resolving the individual lines down to a distance of 150 nm (shown in Fig. S4b). In Fig. S4c, the dip of the individual line pairs is shown for the 100x objective lens. Further, we repeated this evaluation for the 60x objective lens (Fig. S4d) and for the 40x objective lens (Fig. S4e). We are able to show that we can improve the resolution for multiple objective lenses without any additional effort.

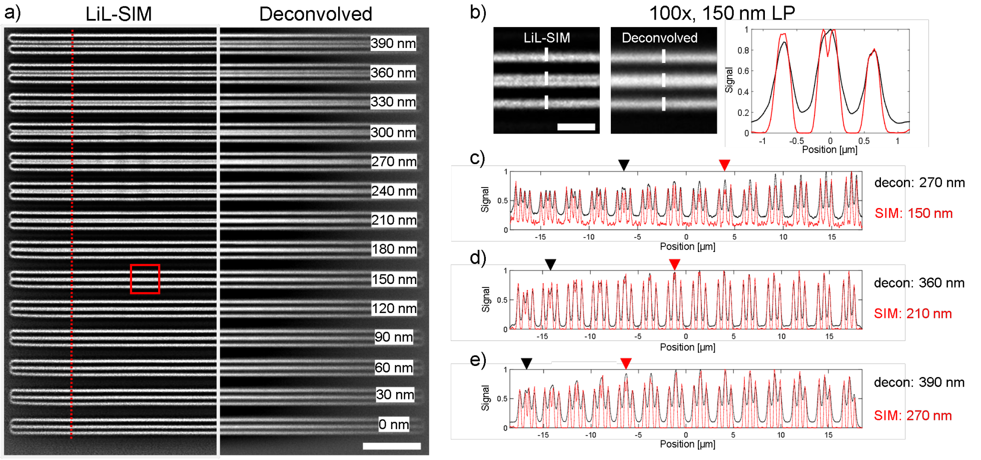

Figure S4 Resolution improvement of LiL-SIM on fluorescent line pairs (LP). a) Comparison of resolvable LP by applying LiL-SIM vs Richardson-Lucy deconvolved LiL-2PM (10 iterations) using a 100x / 1.49 NA objective lens. b) The 150 nm LP is resolved by LiL-SIM, while it cannot be resolved by deconvolved LiL-2PM. c) Line plots along the dashed line in the main image show the resolvable line pairs with the 100x objective lens. The lateral resolution is 270 nm for LiL-2PM and 150 nm for LiL-SIM. d) Corresponding line plot when using the 60x / 1.27 NA objective lens (LiL-2PM resolution: 360 nm, LiL-SIM: 210 nm). e) Corresponding line-plot when using the 40x/ 1.15 NA objective lens (LiL-2PM resolution: 390 nm, LiL-SIM: 270 nm). Scale bars: a) 5 µm, b) 1 µm.

We further evaluated the performance of LiL-SIM with the 60x / 1.27 NA objective lens in *Pinus radiata* (shown in Fig. S5) by comparing WiL-2PM (Fig. S5a and LiL-SIM (Fig. S5b).

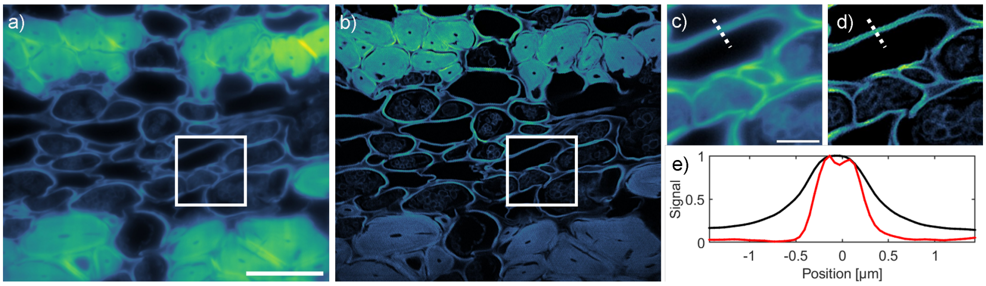

Figure S5 a) WiL-2PM and b) LiL-SIM images of *Pinus radiata* when using a 60x / 1.27 instead of the 100x / 1.49 NA objective lens. This corresponds to a maximum FOV of 111 x 111 µm for the 60x objective lens (compared to a maximum FOV of 67 x 67 µm for the 100x.) c-d) Insets of the white dashed region shown in the main images. e) Line profile comparison along the dashed lines show that the double membrane wall with a distance of 210 nm can be resolved by LiL-SIM while using the lower magnification objective lens with increased FOV. Representative image out of (N = 3) measurements. Scale bars: 20 µm, insets 5 µm.

6 Axial resolution

We compared the axial resolution of PMT-2PM, WiL-2PM and LiL-2PM by measuring the FWHM of the xylem vessel membrane, which has an approx. width of 300-400 nm. The axial resolution of line-scanning and spot-scanning microscopy are equal (both methods overfill the back aperture of the objective lens with a collimated extent, which mainly describes the achieved resolution). However, the detector modality has an influence on lateral and axial resolution. In PMT-2PM, the system PSF can be described by squaring the excitation PSF. However, when switching to camera detection, the system PSF is expressed as a multiplication of the excitation PSF with the emission PSF (which depends on the emission wavelength of the fluorophores). Consequently, both lateral and axial resolution are higher (i.e. exhibit a smaller PSF) when using camera-based detection over point-based detection. In terms of axial resolution, we show this in Fig. S6 by comparing axial xz-cross-sections in *Pinus radiata* tissue. Fig. S6a shows the xz-cross-section when using PMT-2PM, while xz-cross-sections acquired with WiL-2PM and LiL-2PM are depicted in Fig. S6b-c, respectively. The corresponding axial resolutions are 648 ± 32 nm for PMT-2PM, 562 ± 27 nm for WiL-2PM and 508 ± 24 nm for LiL-2PM and LiL-SIM, respectively.

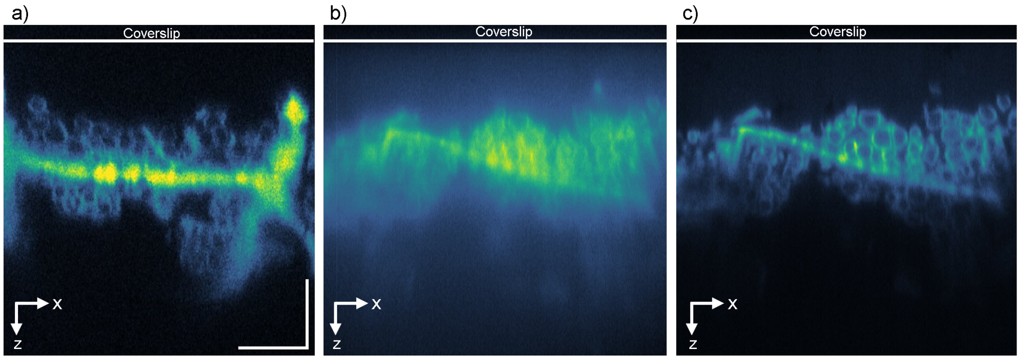

Figure S6 Comparison of xz-cross-sections in *Pinus radiata*, imaged with a) PMT-2PM, b) WiL-2PM and c) LiL-2PM. The axial resolution is increased in the camera-based schemes. Representative images out of (N = 5) measurements.

7 Polarization influence on modulation depth

We studied the influence of polarization on the modulation depth of the illumination patterns. In conventional (interference-based) SIM, azimutal polarizers are used to ensure s-polarization for each pattern orientation. In our case, the illumination pattern is not formed by interference, but theory suggests that depolarization of high NA objectives still significantly alter the extent of the focal spot [2, 3]. Therefore, we generated 389 nm patterns in the fluorescent slide and analyzed the modulation contrast with two different polarization states (perpendicular and parallel). Zoom-ins of the full FOVs are presented in Fig. S7a. We found that the modulation contrast was slightly increased for s-polarized patterns, especially when comparing the modulation along the individual excitation lines (Fig. S7b).

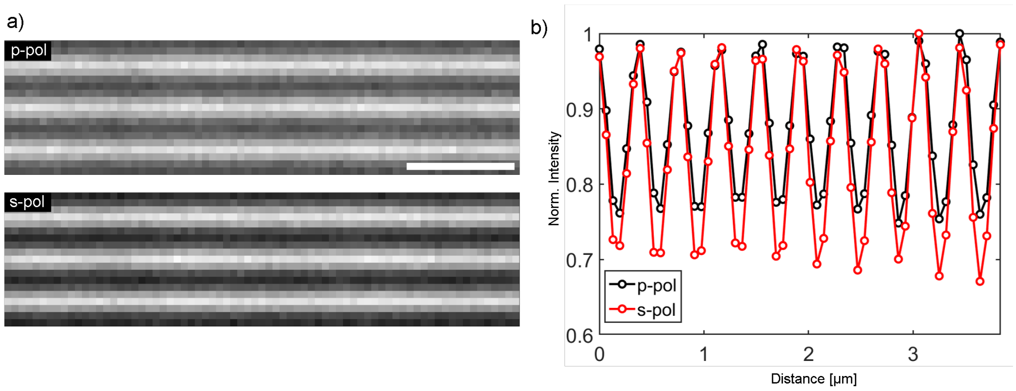

Figure S7 a) Influence of polarization on the resulting illumination patterns. The modulation contrast in the p-polarized pattern is reduced due to depolarization by the high NA objective lens. b) Line plots extracted from both s- and p-polarized images quantify the increase in modulation contrast for s-polarized patterns. Scale bar a) 1 µm.

8 Evaluation of aberrations introduced by the Dove prism

We evaluated the field homogeneity by measuring an evenly distributed array of fluorescent rings (one of the test samples on the Argolight Argo-SIM slide) with our 100x objective lens (see Fig. S8a). No field distortion, nor spherical aberrations were introduced over the entire FOV (65 x 65 µm). We further carried out measurements with a 40x objective lens to cover the full extent of the array of rings. Fig. S8b shows the measurement with the Dove prism inserted in the beam path, while we removed the Dove prism in Fig. S8c. We observed slightly decreased signal intensity when the Dove prism is inserted. This can be explained by pulse broadening due to dispersion. However, the full ROI remained homogeneous, and we could not find any evidence for additional aberrations or field distortion that were introduced by the prism over a FOV of 100 x 100 µm.

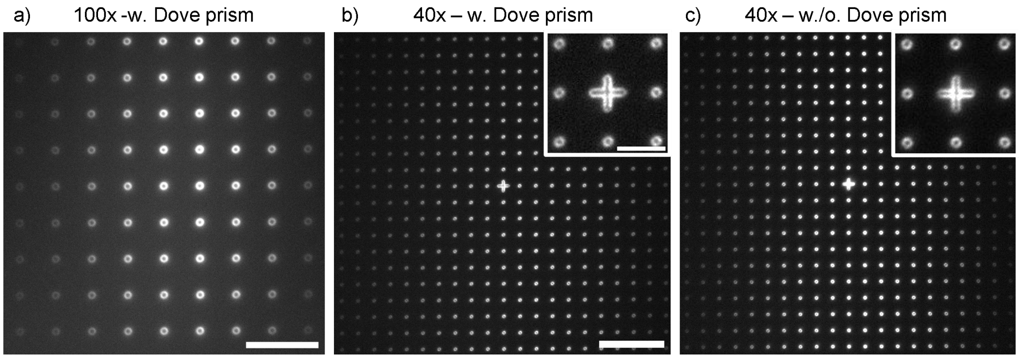

Figure S8 Evaluation of field distortion and aberrations when using the Dove prism in both the excitation and detection beam path. a) Recording the full FOV with the 100x objective lens with inserted Dove prism. b) Recording a FOV of 100 µm x 100 µm using the 40x objective lens (with Dove prism inserted). c) Recording of the same FOV by using the 40x objective lens without the Dove prism. No significant distortions or aberrations were detected. Rings are homogeneous over the entire FOV. Scale bars: a) 10 µm, b) 20 µm, inset 5 µm.

9 Phase stability of the galvo-scanner

As described in the manuscript, the pattern homogeneity depends on the linear movement of the galvo-scanner. Therefore, we carried out measurements to test the phase stability and repeatability of generating fine illumination patterns from 300 to 500 nm. Fig. S9 shows a color-coded representation of the phase shifted patterns acquired with LiL-2PM (red 0°, blue 120°, green 240°) at a pattern spacing of 350 nm. The insets b, c and d show that the phase of the individual patterns remains unaffected over the entire FOV.

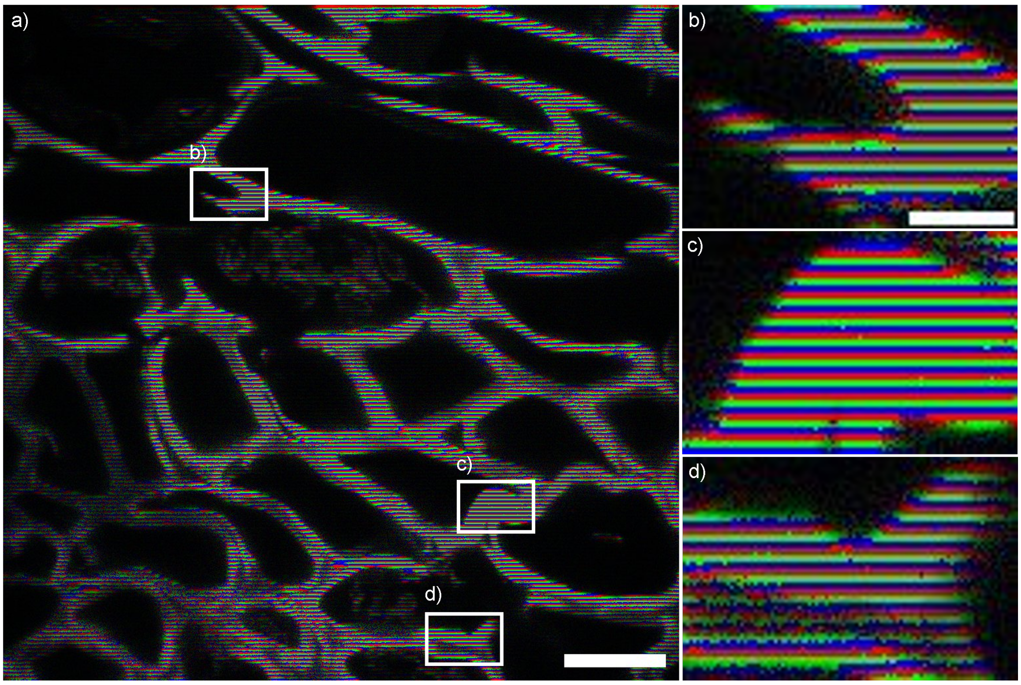

Figure S9 a) Color-coded representation of phase shifted stripe patterns recorded by LiL-2PM (red 0°, blue 120°, green 240°). b-d) Insets of three regions from the main image. The phase of the individual patterns remains stable over the entire FOV. Scale bars: a) 10 µm, b-d) 2 µm.

10 LiL-SIM with three / five patterns per orientation

We carried out additional measurements to analyze the difference between three and five patterns per orientation, visualized in Fig. S10. Recording only three patterns per orientation speeds up the imaging process by a factor of 1.6 but lowers the overall SNR. We also observed minor stripe artefacts, that are present in the images in LiL-SIM images with three patterns per orientation. Since we were not measuring dynamic processes in the experiments shown in the manuscript, we decided to use five patterns per orientation for an improved SNR at deep tissue sections.

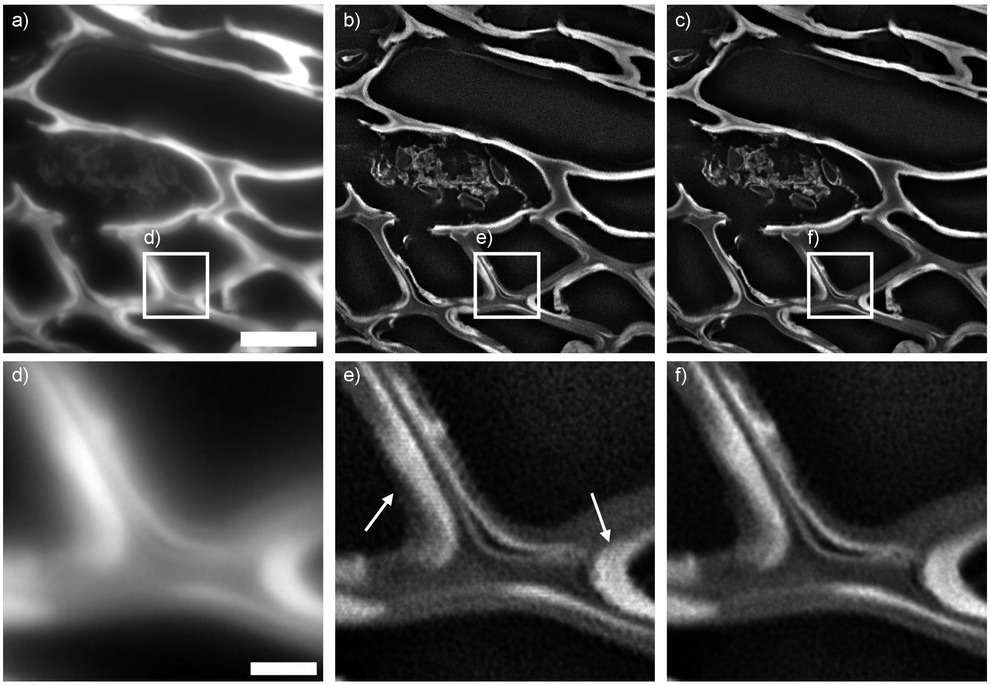

Figure S10 Comparison between a) WiL-2PM b) LiL-SIM with three patterns per orientation and c) LiL-SIM with five patterns per orientation. The SNR with five patterns is increased. Minor stripe artefacts are present in the three pattern images (indicated by the white arrows). Representative images out of (N = 3) measurements. Scale bars a-c) 10 µm, d-f) 2 µm.

11 Simulations for determining the resolution improvement factor in LiL-SIM

Simulations have been performed to demonstrate the imaging process and resolution improvement of LiL-SIM. The script handles the generation of the Point Spread Function (PSF) according to the Gibson-Lanni model [4], scanning routines with either beads or a homogeneous fluorescent surface, and noise modeling to simulate realistic imaging conditions in deep tissue. The workflow of the simulation is shown in Fig. S11a. After generating the images, the open-source software fairSIM is used to reconstruct the super-resolved images. We ran reconstructions with various pattern periods ranging from 250 to 600 nm on simulated bead images, shown in Fig. S11b and determined a maximum resolution improvement factor of 1.84 compared to the Rayleigh-limit. However, this factor might not be reached in deep tissue layers since the decrease of SNR and aberrations degrade the modulation contrast. From our experimental results, we mostly used pattern spacings from 300 to 350 nm, indicated by the red box. Based on our simulation results, this corresponds to achievable resolution factors of 1.61 - 1.74 in relation to the Rayleigh-limit.

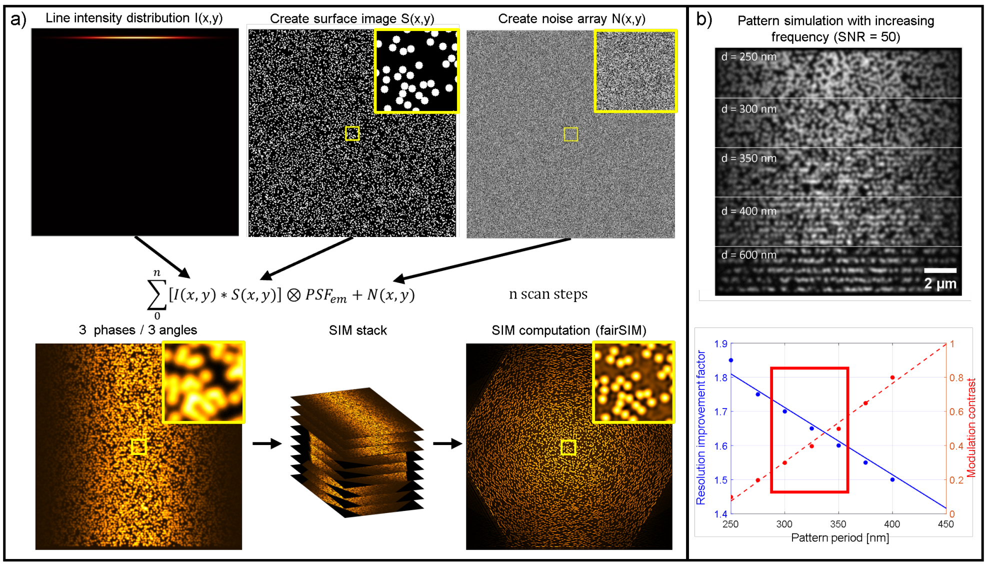

Figure S11 a) Workflow for LiL-SIM simulation. A line-intensity distribution I(x,y) is generated and multiplied with surface image S(x,y). For each scan step, Gaussian-Poisson noise is added. The sequential scanning procedure is repeated with three initial staring phases at three rotation angles to generate a SIM-computible image stack. Subsequent SIM reconstruction by fairSIM increases the resolution and resolves 190 nm beads which were not resolved in the averaged image. b) Simulation of pattern periods from 250 - 600 nm to determine the maximum resolution improvement factor of LiL-SIM. In aberration- and noise-free image conditions, the maximum resolution improvement factor is 1.84. When introducing noise to the simulation, the resolution improvement factors ranged from 1.61 to 1.74 compared to the Rayleigh-limit, indicated by the red box.

12 Lightsheet shutter (LSS) mode enhances pattern contrast in scattering samples

Fig. S12a demonstrates the increase in pattern contrast when using LSS over RS mode in fluorescent slides, composed of highly concentrated fluorophores. Fig. S12b shows the decrease of pattern contrast with increasing imaging depth. Here, it is important to state that we analyze the modulation depth of the recorded fluorescent patterns, and not the modulation depth of the excitation pattern, which should lie between 70 and 100 percent for achieving high contrast in the recorded patterns. The initial modulation contrast value of the 400 nm pattern recorded with RS mode (dashed curve) is substantially lowered compared to the patterns recorded with LSS mode. The exposure time of each pixel row was set to 5 ms while the speed of the exposure band was set to one line per 0.8 ms. This results in an imaging time of 415 ms for an area of 512x512 pixels. Image acquisition time can be further improved by using more sensitive cameras (EMCCD or APD arrays).

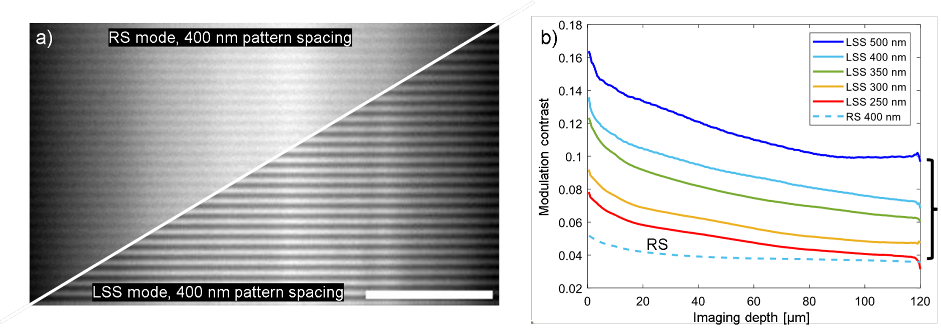

Figure S12 a) Comparison of 400 nm patterns generated with LSS and RS mode in thick fluorescent slides. b) Modulation contrast of various patterns as a function of imaging depth in fluorescent slides with pattern periods ranging from 250 to 500 nm. The modulation contrast decreases with increasing imaging depth when using LSS mode (solid curves) and is substantially decreased when using RS mode (dashed curve). Scale bars: a) 5 µm.

13 Determination of the diffraction limited resolution in LiL-SIM

The vertical extent of the line-excitation profile is diffraction-limited and can be calculated by Eq. 1 [5].

$$d_{FWHM} = \frac{0.325\lambda}{\sqrt{2}\mathrm{NA}^{0.91}} \cdot 2\sqrt{\ln 2} \qquad \mathrm{NA} > 0.7 \tag{1}$$

The experimental diffraction-limited resolution determined from the bead measurement is approx. 275 nm at an emission wavelength of 520 nm (NA = 1.49).

The calculated Abbe-limit with the 1.49 NA objective would be 174 nm. Those theoretical values of resolution are almost never reached, especially not when imaging dense samples (due to aberrations and scattering in dense tissue). For proving this, we looked at the most common two-photon SIM literature [6–9] and compared their achieved resolutions with theoretical values. The results are visualized in Tab. T3.

Overview of diffraction limited resolution in two-photon SIM literature						
Author	λ_{ex} [nm]	λ_{em} [nm]	NA	d_{exp} [nm]	d_{theo} [nm]	d_{SIM} [nm]
Winter2014	900	515	1.2	311 ± 10	223	145 ± 5
Ingaramo2014	930	525	1.2	360 ± 30	219	160 ± 20
Li2017	800	480	1.0	410	240	208
Zhang2023	920	525	1.1	349 ± 21	238	142 ± 11

Table T3 Comparison of experimental diffraction limit and theoretical resolution limit in two-photon SIM literature.

14 Flat-field correction of LiL-SIM images

The intensity decay of the images due to the geometry of the excitation profile is compensated by flatfield correction (Fig. S13).

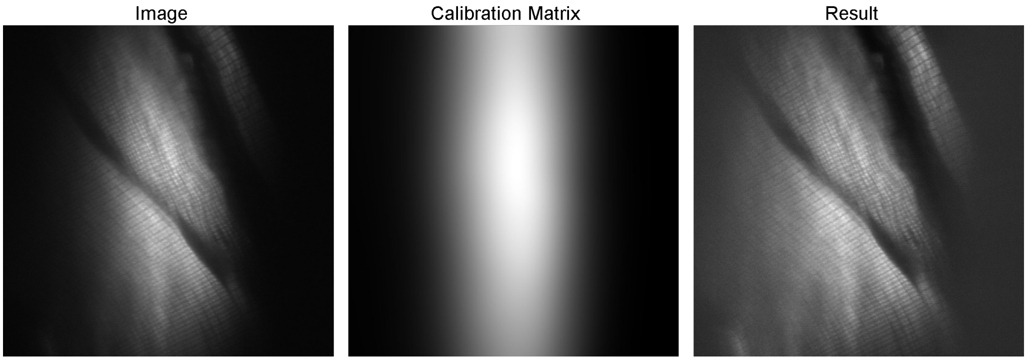

Figure S13 Imaging of sarcomeres from the zebrafish sample. The line excitation profile has a Gaussian envelope, which leads to an intensity decay dependent of the position from the center. The intensity decay can be corrected by recording the calibration matrix. Image divided by the calibration matrix yields a corrected image with increased FOV.

15 Script for digital back rotation and stitching of LiL-2PM image sets

If publicly available SIM algorithms should be used to reconstruct the LiL-SIM image, the images recorded with 60° and 120° rotation need to undergo digital back

rotation, explained in section 2.2 of the manuscript. Therefore, we implemented an automated process to align LiL-SIM image stacks in prior to the SIM reconstruction. The script automatically determines the rotational and translational shifts from the images based on either phase correlation (PC) image registration [10] or Scale-Invariant Feature Transform (SIFT) [11]. The choice of reconstruction algorithm depends on the strength of intensity gradients in the raw images. Optionally, the user can chose for pseudo-flat field correction or flat field correction based on the calibration matrix if the illumination distribution is known (see. S13).

16 Decorrelation curves of bead and *Pinus radiata* measurement

Resolution is estimated by computing the images with image decorrelation analysis [12], a tool that analyzes the highest spatial frequencies present in the image. Representative decorrelation curves of beads (Fig. 1c) and *Pinus radiata* measurements (Fig. 3) are visualized in Fig. S14.

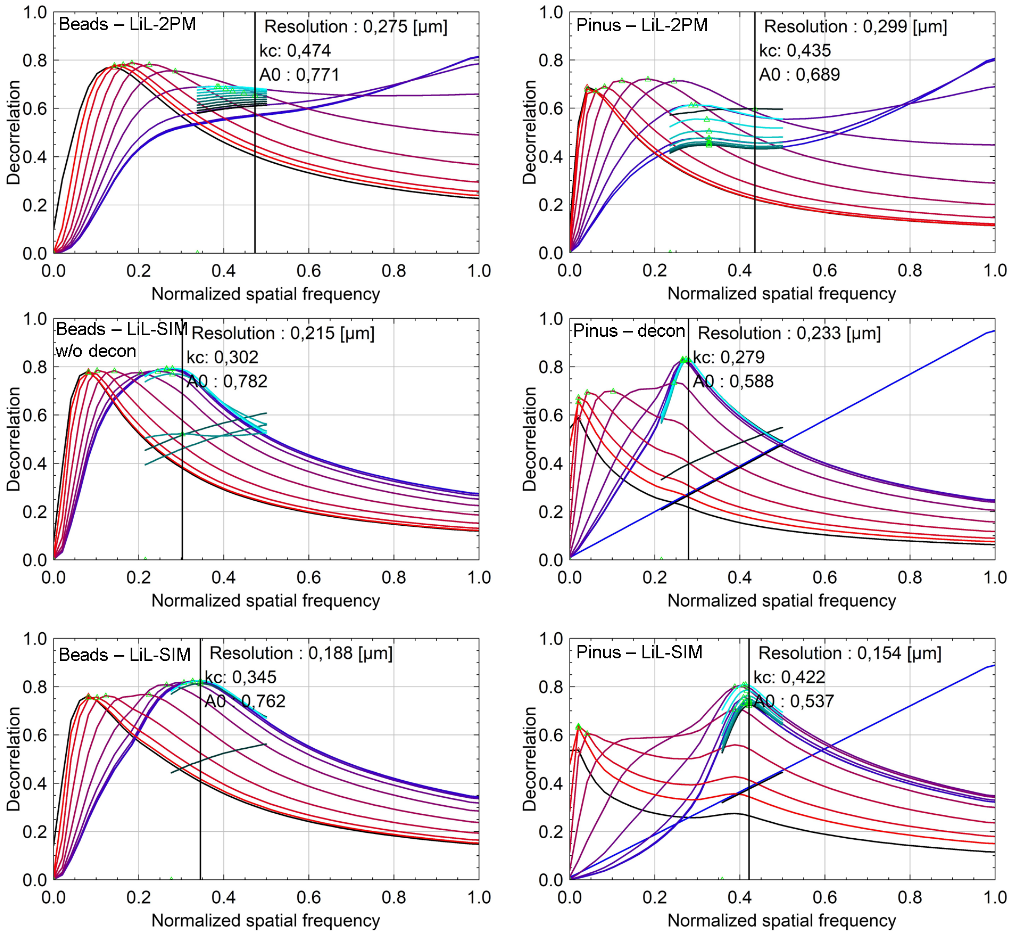

Figure S14 Image decorrelation curves of the LiL-2PM, deconvolved LiL-2PM and LiL-SIM images of the (left) bead and (right) *Pinus radiata* measurement shown in manuscript Fig. 5,6a.

17 Comparison of super-resolution methods

We compare state-of-the-art super-resolution methods based on the lateral and axial resolution (Fig. S15a), as well as on the penetration depth (Fig. S15b). The lateral resolution of single-molecule localization microscopy (SMLM) is typically superior compared to deterministic methods such as SIM and STED. It is, however, not only the spatial resolution that can be achieved which is important, but the penetration depth to which this resolution can be maintained is also relevant for biological applications. In Fig. S15b, below, we compare the imaging depth of SRM methods based on their penetration capability. As shown in this figure, the penetration depth of any imaging modality can be substantially increased if 2P fluorescence excitation (red bars) is used instead of 1P excitation (black bars). Starting with the methods using 1P excitation, it can be seen that 1P confocal microscopy is currently the only method which can achieve imaging depths of about 100 µm [13] in native samples (i.e. samples that are not fixed and optically cleared). 1P SIM is mostly restricted to around 15 µm depth [14] because of the degradation of the modulation contrast in deeper sample planes. 1P STED is also limited to around 15 µm [15] due to the distortions of the STED beam caused by aberrations and scattering. However, implementations of adaptive optics can further increase the imaging depth of 1P SIM up to values of 50 µm [16]. Most localization-based methods are basically restricted to imaging at the coverslip surface. Some approaches to deep imaging using 3D-STORM and 3D-SOFI have been made, but the penetration depth doesn't exceed approx. 6 µm [17–20]. Vaziri et al demonstrated localization microscopy at depths of up to 10 µm by using two-photon temporal focusing for fluorescence excitation [21]. More recently, the group of

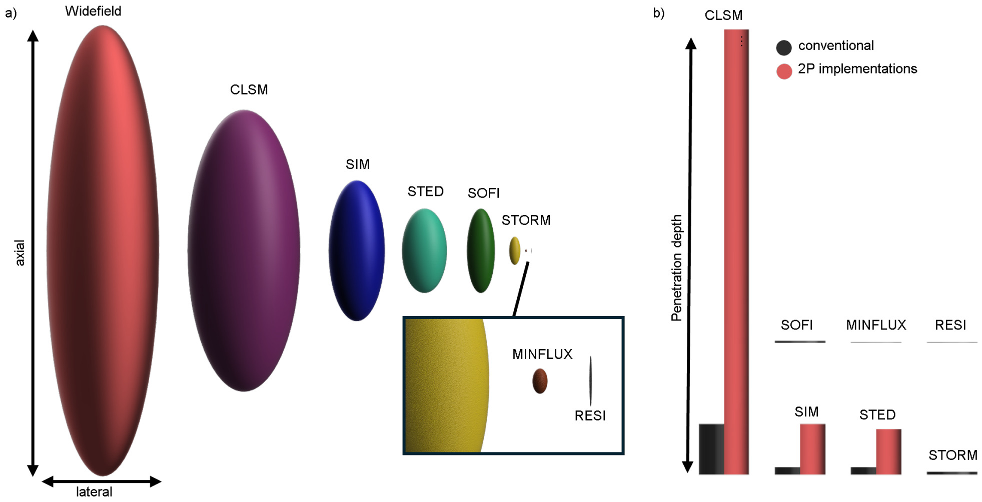

Figure S15 Comparison of SRM methods based on a) lateral and axial resolution and b) penetration depth. The localization methods have far superior lateral and axial resolution. b) Penetration depths of 1P implementations (black) and 2P implementations (red). While CLSM, SIM and STED can be used for deep tissue imaging, the localization based methods are restricted to imaging close to the coverslip.

Ralf Jungmann demonstrated imaging of cells with DNA-PAINT in combination with spinning disk confocal microscopy of entire cells in depths of up to 10 µm [22]. Also, recently two-photon-excited STED has been combined with adaptive optics (AO) and was demonstrated at imaging depths of up to 90 µm [23, 24]. The implementations of 2P-SIM accomplished imaging depths of up to 110 µm [6, 7] even without the need for AO. For further information on the temporal speed of these methods, we refer to excellent reviews [25–27].

18 Parts list

Part	Model/Vendor	Cost
**Optical setup**		
Laser	Toptica prototype	-
Cylindrical lens	**LJ1695RM-B / Thorlabs**	**109.05 €**
Scanner	GVS002 / Thorlabs	2,134.28 €
Scan lens	SL50-CLS2 / Thorlabs	3,250.27 €
Scanning tube lens	TTL200MP / Thorlabs	1,382.43 €
Piezoelectric rotation mount	**ELL14K / Thorlabs**	**545.76 €**
Half-wave plate	**WPMS05-808 / Thorlabs**	**461.09 €**
Dove prism	**PS992M-A / Thorlabs**	**241.88 €**
Objective lens	CFI Apochromat TIRF 100XC Oil / Nikon	-
**Dichroic and Filters**		
Dichroic	F76-705 / AHF	≈ 600 €
Cleanup filter	F75-680 / AHF	-
Detection filter 1	F37-630 / AHF	-
Detection filter 2	F37-584 / AHF	≈ 400 €
Detection tube lens	AC254-200-A-ML / Thorlabs	105.83 €
**Camera**		
Camera	**Panda 4.2 / PCO**	≈ **8000 €**
**Hardware**		
DAQ Card	PCIe 6341 / NI	≈ 1300 €
Breakout box	BNC2110 / NI	≈ 700 €
Z-stage	MT1/M-Z8, KDC101 / Thorlabs	≈ 1000 €
XY-stages	KMTS50E/M / Thorlabs	1,687.34 €
XYZ-hub controller	KCH301 / Thorlabs	≈ 600 €

Table T4 LiL-SIM optical setup and hardware components. The models marked in bold need to be purchased to implement the LiL-SIM method into a conventional 2P microscope.

Components used to build the microscope are briefly collected in the parts list. Models marked in bold need to be additionally purchased to implement the LiL-SIM method into a conventional 2P microscope. This amounts to a total of below 10.000 € for the modification (including the sCMOS camera). We added this parts list in the Supplementary document (T4). Furthermore, we added a reference to the laser source in the main manuscript.

References

[1] Stuurman, N., Amdodaj, N., Vale, R.: µ-manager: open source software for light microscope imaging. Microscopy Today **15**(3), 42–43 (2007)

[2] B, R., E, W.: Electromagnetic diffraction in optical systems, ii. structure of the image field in an aplanatic system. Proceedings of the Royal Society of London. Series A. Mathematical and Physical Sciences **253**, 358–379 (1959) https://doi.org/10.1098/rspa.1959.0200

[3] Dorn, R., Quabis, S., Leuchs, G.: The focus of light—linear polarization breaks the rotational symmetry of the focal spot. Journal of Modern Optics **50**, 1917–1926 (2003) https://doi.org/10.1080/09500340308235246

[4] Gibson, S.F., Lanni, F.: Experimental Test of an Analytical Model of Aberration in an Oil-immersion Objective Lens Used in Three-dimensional Light Microscopy

[5] Zipfel, W.R., Williams, R.M., Webb, W.W.: Nonlinear magic: Multiphoton microscopy in the biosciences. Nature Biotechnology **21**, 1369–1377 (2003) https://doi.org/10.1038/nbt899

[6] Winter, P.W., York, A.G., Nogare, D.D., Ingaramo, M., Christensen, R., Chitnis, A., Patterson, G.H., Shroff, H.: Two-photon instant structured illumination microscopy improves the depth penetration of super-resolution imaging in thick scattering samples. Optica **1**, 181 (2014) https://doi.org/10.1364/optica.1.000181

[7] Ingaramo, M., York, A.G., Wawrzusin, P., Milberg, O., Hong, A., Weigert, R., Shroff, H., Patterson, G.H.: Two-photon excitation improves multifocal structured illumination microscopy in thick scattering tissue. Proceedings of the National Academy of Sciences of the United States of America **111**, 5254–5259 (2014) https://doi.org/10.1073/pnas.1314447111

[8] Li, Z., Hou, J., Suo, J., Qiao, C., Kong, L., Dai, Q.: Contrast and resolution enhanced optical sectioning in scattering tissue using line-scanning two-photon structured illumination microscopy. Optics Express **25**, 32010 (2017) https://doi.org/10.1364/oe.25.032010

[9] Zhang, C., Yu, B., Lin, F., Samanta, S., Yu, H., Zhang, W., Jing, Y., Shang, C., Lin, D., Si, K., Gong, W., Qu, J.: Deep tissue super-resolution imaging with adaptive optical two-photon multifocal structured illumination microscopy. PhotoniX **4**, 38 (2023) https://doi.org/10.1186/s43074-023-00115-2

[10] Kuglin, C.D.: The phase correlation image alignment method. In: IEEE Int. Conf. on Cybernetics and Society, 1975, pp. 163–165 (1975)

[11] Lowe, D.G.: Distinctive image features from scale-invariant keypoints. International Journal of Computer Vision **60**, 91–110 (2004) https://doi.org/10.1023/B:VISI.0000029664.99615.94

[12] Descloux, A., Grußmayer, K.S., Radenovic, A.: Parameter-free image resolution estimation based on decorrelation analysis. Nature Methods **16**, 918–924 (2019) https://doi.org/10.1038/s41592-019-0515-7

[13] Sahu, P., Mazumder, N.: Improving the Way We See: Adaptive Optics Based Optical Microscopy for Deep-Tissue Imaging. https://doi.org/10.3389/fphy.2021.654868

[14] Heintzmann, R., Huser, T.: Super-Resolution Structured Illumination Microscopy. https://doi.org/10.1021/acs.chemrev.7b00218 . limited imaging depth to 15 µm

[15] Berning, S., Willig, K.I., Steffens, H., Dibaj, P., Hell, S.W.: Nanoscopy in a Living Mouse Brain. https://doi.org/10.1126/science.1215369

[16] Turcotte, R., Liang, Y., Tanimoto, M., Zhang, Q., Li, Z., Koyama, M., Betzig, E., Ji, N.: Dynamic super-resolution structured illumination imaging in the living brain. Proceedings of the National Academy of Sciences **116**, 9586–9591 (2019) https://doi.org/10.1073/pnas.1819965116

[17] Dani, A., Huang, B., Bergan, J., Dulac, C., Zhuang, X.: Superresolution imaging of chemical synapses in the brain. Neuron **68**, 843–856 (2010) https://doi.org/10.1016/j.neuron.2010.11.021

[18] Herrmannsdörfer, F., Flottmann, B., Nanguneri, S., Venkataramani, V., Horstmann, H., Kuner, T., Heilemann, M.: 3d d storm imaging of fixed brain tissue. Methods in Molecular Biology **1538**, 169–184 (2017) https://doi.org/10.1007/978-1-4939-6688-2_13

[19] Dertinger, T., Colyer, R., Iyer, G., Weiss, S., Enderlein, J.: Fast, background-free, 3d super-resolution optical fluctuation imaging (sofi). Proceedings of the National Academy of Sciences **106**, 22287–22292 (2009) https://doi.org/10.1073/pnas.0907866106

[20] Dertinger, T., Xu, J., Naini, O.F., Vogel, R., Weiss, S.: Sofi-based 3d superresolution sectioning with a widefield microscope. Optical Nanoscopy **1**, 1–5 (2012) https://doi.org/10.1186/2192-2853-1-2

[21] Vaziri, A., Tang, J., Shroff, H., Shank, C.V.: Multilayer Three-dimensional Super Resolution Imaging of Thick Biological Samples. www.pnas.org/cgi/content/full/0810636105/DCSupplemental.www.pnas.orgcgidoi10.1073pnas.0810636105

[22] Schueder, F., Lara-Gutiérrez, J., Beliveau, B.J., Saka, S.K., Sasaki, H.M., Woehrstein, J.B., Strauss, M.T., Grabmayr, H., Yin, P., Jungmann, R.: Multiplexed 3d super-resolution imaging of whole cells using spinning disk confocal microscopy and dna-paint. Nature Communications **8** (2017) https://doi.org/10.1038/s41467-017-02028-8

[23] Velasco, M.G.M., Zhang, M., Antonello, J., Yuan, P., Allgeyer, E.S., May, D., M'Saad, O., Kidd, P., Barentine, A.E.S., Greco, V., Grutzendler, J., Booth, M.J., Bewersdorf, J.: 3d super-resolution deep-tissue imaging in living mice. Optica **8**, 442 (2021) https://doi.org/10.1364/optica.416841

[24] Bancelin, S., Mercier, L., Murana, E., Nägerl, U.V.: Aberration correction in stimulated emission depletion microscopy to increase imaging depth in living brain tissue. Neurophotonics **8** (2021) https://doi.org/10.1117/1.nph.8.3.035001

[25] Winter, P.W., Shroff, H.: Faster fluorescence microscopy: Advances in high speed biological imaging. Current Opinion in Chemical Biology **20**, 46–53 (2014) https://doi.org/10.1016/j.cbpa.2014.04.008

[26] Godin, A.G., Lounis, B., Cognet, L.: Super-resolution Microscopy Approaches for Live Cell Imaging. https://doi.org/10.1016/j.bpj.2014.08.028

[27] Schermelleh, L., Ferrand, A., Huser, T., Eggeling, C., Sauer, M., Biehlmaier, O., Drummen, G.P.C.: Super-resolution Microscopy Demystified. https://doi.org/10.1038/s41556-018-0251-8
