# Supplementary material for: Super-resolution upgrade for deep tissue imaging featuring simple implementation
Source: Nat Commun. 2025 Jun 25;16:5386. doi: 10.1038/s41467-025-60744-y (PMC12198360; doi:10.1038/s41467-025-60744-y)
Supplement: Supplementary file 2 — Description of Additional Supplementary Files [file 41467_2025_60744_MOESM2_ESM.docx]

**Description of Additional Supplementary Files**

**Supplementary Movie 1**:

The volume imaging capability of LiL-2PM in thick scattering specimens is shown in Video1. Consecutive planes have been recorded by using LiL-2PM with an axial step width of 0.5 µm, leading to a highresolution volume image of the structure. It is clearly visible that the implementation of LSS mode in line-scanning microscopy enhances the contrast of the acquired volume image compared to WiL-2PM. Speaking of resolution, it is important to state that LiL-2PM does not yield a resolution gain itself compared to diffraction limited WiL-2PM imaging. However, in samples with dense fluorophore concentration, resolution in WiL-2PM might be lowered due to the blur introduced by scattered photons.

**Supplementary Movie 2:**

In order to demonstrate the temporal resolution of LiL-SIM, we lowered the line integration time to 1 ms and performed SIM reconstructions along a single orientation on the Pinus radiata sample (see Video2). The integration time for a single frame is set to 76 ms (including the flyback array), covering an area of 512 x 512 pixels. If three phases are recorded, the acquisition time is 228 ms for a single orientation
