# Supplementary material for: Super-resolution upgrade for deep tissue imaging featuring simple implementation
Source: Nat Commun. 2025 Jun 25;16:5386. doi: 10.1038/s41467-025-60744-y (PMC12198360; doi:10.1038/s41467-025-60744-y)
Supplement: Supplementary file 5 — Reporting Summary [file 41467_2025_60744_MOESM5_ESM.pdf]

nature portfolio

Corresponding author(s): Thomas Hellerer

Last updated by author(s): Mar 19, 2025

Reporting Summary

Nature Portfolio wishes to improve the reproducibility of the work that we publish. This form provides structure for consistency and transparency in reporting. For further information on Nature Portfolio policies, see our Editorial Policies and the Editorial Policy Checklist.

Statistics

For all statistical analyses, confirm that the following items are present in the figure legend, table legend, main text, or Methods section.

n/a	Confirmed	
[ ]	[x]	The exact sample size (*n*) for each experimental group/condition, given as a discrete number and unit of measurement
[ ]	[x]	A statement on whether measurements were taken from distinct samples or whether the same sample was measured repeatedly
[x]	[ ]	The statistical test(s) used AND whether they are one- or two-sided<br>*Only common tests should be described solely by name; describe more complex techniques in the Methods section.*
[x]	[ ]	A description of all covariates tested
[x]	[ ]	A description of any assumptions or corrections, such as tests of normality and adjustment for multiple comparisons
[ ]	[x]	A full description of the statistical parameters including central tendency (e.g. means) or other basic estimates (e.g. regression coefficient) AND variation (e.g. standard deviation) or associated estimates of uncertainty (e.g. confidence intervals)
[x]	[ ]	For null hypothesis testing, the test statistic (e.g. *F*, *t*, *r*) with confidence intervals, effect sizes, degrees of freedom and *P* value noted<br>*Give P values as exact values whenever suitable.*
[x]	[ ]	For Bayesian analysis, information on the choice of priors and Markov chain Monte Carlo settings
[x]	[ ]	For hierarchical and complex designs, identification of the appropriate level for tests and full reporting of outcomes
[x]	[ ]	Estimates of effect sizes (e.g. Cohen's *d*, Pearson's *r*), indicating how they were calculated

Our web collection on statistics for biologists contains articles on many of the points above.

Software and code

Policy information about availability of computer code

Data collection	The microscope was controlled with custom-written Matlab software (Matlab R2022b). The camera settings were controlled in Micromanager 2.0. The acquired raw images were post-processed by custom-written Python software (Anaconda 3.11.5).
Data analysis	Data was analyzed with custom-written scripts (Matlab R022b) and Fiji (ImageJ 1.54f). SIM images were calculated by using the open-source plugin fairSIM (ImageJ implementation, git build id: 584010c43 standard build). For resolution estimation, the Fourier Ring correlation plugin (BIOP ImageJ implementation 1.0.2) and decorrelation analysis (ImageJ implementation v1.1.8) were used.

For manuscripts utilizing custom algorithms or software that are central to the research but not yet described in published literature, software must be made available to editors and reviewers. We strongly encourage code deposition in a community repository (e.g. GitHub). See the Nature Portfolio guidelines for submitting code & software for further information.

Data

Policy information about availability of data

All manuscripts must include a data availability statement. This statement should provide the following information, where applicable:

- Accession codes, unique identifiers, or web links for publicly available datasets
- A description of any restrictions on data availability
- For clinical datasets or third party data, please ensure that the statement adheres to our policy

Detailed wiring diagrams, technical notes, as well as a parts list to rebuild the LiL-SIM setup are available in the supplemental document and in Github repository at https://doi.org/10.5281/zenodo.15031580. Raw data of the acquired volumes, raw SIM reconstruction data and the reconstructed SIM image set (which also

includes the widefield and the deconvolved images) for Figures 1-4 of the main manuscript is available at https://doi.org/10.5281/zenodo.15031504. Source data is provided with this paper. Data underlying the results presented in the supplemental document exceeds the capacity of the repository but is available upon request.

Research involving human participants, their data, or biological material

Policy information about studies with human participants or human data. See also policy information about sex, gender (identity/presentation), and sexual orientation and race, ethnicity and racism.

Reporting on sex and gender	n.a.
Reporting on race, ethnicity, or other socially relevant groupings	n.a.
Population characteristics	n.a.
Recruitment	n.a.
Ethics oversight	n.a.

Note that full information on the approval of the study protocol must also be provided in the manuscript.

Field-specific reporting

Please select the one below that is the best fit for your research. If you are not sure, read the appropriate sections before making your selection.

☒ Life sciences ☐ Behavioural & social sciences ☐ Ecological, evolutionary & environmental sciences

For a reference copy of the document with all sections, see nature.com/documents/nr-reporting-summary-flat.pdf

Life sciences study design

All studies must disclose on these points even when the disclosure is negative.

Sample size	SIM images presented in Fig. 1c, 2d, 4 were successfully reconstructed at (N = 5 times) distinct but closely neighbouring positions at same imaging depth with similar resolution improvements (repeatedly measured on the same sample). SIM images presented in Fig. 3 have been successfully reconstructed (N = 10 times) per imaging depth. Volume stacks presented in Fig. 2d, 3a, 4 have been acquired (N = 3) times. Standard deviations of lateral and axial resolution were reported with the mean values in the main text of the manuscript.
Data exclusions	In Fig. 4, we excluded planes from 20-30 µm because no structures were found in those and intermediate planes. We added the stacks including those planes in our Zenodo reposititory, available under https://doi.org/10.5281/zenodo.15031504.
Replication	We repeatedly used the same samples of Pinus radiata, zebrafish and heart muscle, anticipating consistent results in resolution gain and imaging quality (which we can verify in over N>=300 measurements). SIM reconstructions presented in the manuscript were performed at least N = 5 times per imaging depth, while volume stacks were recorded at least N= 3 times due to their larger file size. We don’t expect any deviation in the presented results after evaluating data with the specified sample sizes.
Randomization	Randomization is not relevant for this study since we were solely focusing on demonstrating the super-resolution capability in various deep tissue samples. Samples were therefore not allocated into different experimental groups.
Blinding	Blinding to group allocation was not relevant to our study, as we focused solely on demonstrating a super-resolution method in deep tissue layers. Our analysis was limited to imaging performance rather than the biological relevance of the measured samples.

Reporting for specific materials, systems and methods

We require information from authors about some types of materials, experimental systems and methods used in many studies. Here, indicate whether each material, system or method listed is relevant to your study. If you are not sure if a list item applies to your research, read the appropriate section before selecting a response.

Materials & experimental systems

n/a	Involved in the study
☒	☐ Antibodies
☒	☐ Eukaryotic cell lines
☒	☐ Palaeontology and archaeology
☐	☒ Animals and other organisms
☒	☐ Clinical data
☒	☐ Dual use research of concern
☐	☒ Plants

Methods

n/a	Involved in the study
☒	☐ ChIP-seq
☒	☐ Flow cytometry
☒	☐ MRI-based neuroimaging

Animals and other research organisms

Policy information about studies involving animals; ARRIVE guidelines recommended for reporting animal research, and Sex and Gender in Research

Laboratory animals	The C57BL/6 mouse used in this study is a wild-type strain of Mus musculus. This inbred strain is known for its genetic uniformity, which helps minimize variability in experimental results. The mice were male and aged 16 weeks. It was bred in-house.. The mouse was housed under controlled environmental conditions. It was maintained on a 12-hour light/dark cycle regulated by timers. The cage was supplied with air through an air conditioning system to ensure proper ventilation. The temperature was kept between 20-22°C, and the humidity was maintained at 45-60% to provide optimal living conditions.
Wild animals	This study did not involve the use of material obtained from wild animals.
Reporting on sex	As stated above, sex is not relevant for this study.
Field-collected samples	This study did not involve samples collected from the field.
Ethics oversight	The animal experiments were approved by Regierung von Oberbayern, Sachgebiet 54 – Verbraucherschutz und Veterinärwesen under the license ROB-55.2-2532.Vet_02-18-177. All animal procedures were conducted in accordance with ethical guidelines and were approved by the Regierung von Oberbayern (Government of Bavaria, Germany). The study was conducted under license number Organentnahme §4-Schunkert. For further information, the responsible authority can be contacted at Sachgebiet 54 – Verbraucherschutz, Veterinärwesen, Maximilianstraße 39, 80538 München, Germany.

Note that full information on the approval of the study protocol must also be provided in the manuscript.

Dual use research of concern

Policy information about [dual use research of concern]

Hazards

Could the accidental, deliberate or reckless misuse of agents or technologies generated in the work, or the application of information presented in the manuscript, pose a threat to:

No	Yes	
☒	☐	Public health
☒	☐	National security
☒	☐	Crops and/or livestock
☒	☐	Ecosystems
☒	☐	Any other significant area

Experiments of concern

Does the work involve any of these experiments of concern:

No	Yes	
☒	☐	Demonstrate how to render a vaccine ineffective
☒	☐	Confer resistance to therapeutically useful antibiotics or antiviral agents
☒	☐	Enhance the virulence of a pathogen or render a nonpathogen virulent
☒	☐	Increase transmissibility of a pathogen
☒	☐	Alter the host range of a pathogen
☒	☐	Enable evasion of diagnostic/detection modalities
☒	☐	Enable the weaponization of a biological agent or toxin
☒	☐	Any other potentially harmful combination of experiments and agents

Plants

Seed stocks	The Pinus radiata sample is a commercial product, available at Catalog No. 5986003, Bresser
Novel plant genotypes	No novel plant genotypes were used in this study.
Authentication	This section is not applicable to our study, as we did not use any novel plant genotypes or conduct experiments related to mutation effects or secondary genetic changes.
